# *Addendum:* Pardo, J.; Zamora-Martínez, F.; Botella-Rocamora, P. Online Learning Algorithm for Time Series Forecasting Suitable for Low Cost Wireless Sensor Networks Nodes. *Sensors* 2015, *15*, 9277–9304

**DOI:** 10.3390/s150716831

**Published:** 2015-07-13

**Authors:** Juan Pardo, Francisco Zamora-Martínez, Paloma Botella-Rocamora

**Affiliations:** ESAI—Embedded Systems and Artificial Intelligence Group, Escuela Superior de Enseñanzas Técnicas, Universidad CEU Cardenal Herrera, C/San Bartolomé, 46115 Alfara del Patriarca, Valencia, Spain: E-Mails: francisco.zamora@uch.ceu.es (F.Z.-M.); pbotella@uch.ceu.es (P.B.-R.)

The authors wish to update the Acknowledgments in their paper published in *Sensors* [1], doi:10.3390/s150409277, website: http://www.mdpi.com/1424-8220/15/4/9277.
